# Impact of leadless pacemaker implantation site on cardiac synchronization and tricuspid regurgitation

**DOI:** 10.1186/s43044-024-00602-2

**Published:** 2025-01-06

**Authors:** Xiao-fei Gao, Hong Zhu, Jia-sheng Zhang, Ning-Zhang, Xiao-hong Pan, Yi-Zhou Xu

**Affiliations:** 1 Department of Cardiology, Hangzhou First People’s Hospital, #261 Huansha Road, Hangzhou, 310000 Zhejiang Province China; 2 Department of Cardiology, The Second Affiliated Hospital Zhejiang University School of Medicine, Hangzhou, Zhejiang Province China

**Keywords:** Leadless pacemaker, Synchronization, Pacemaker-induced cardiomyopathy

## Abstract

**Background:**

To investigate the optimization of leadless pacemaker placement and to assess its impact on heart synchronization and tricuspid regurgitation.

**Results:**

A clinical trial was conducted involving 53 patients who underwent leadless pacemaker implantation at the Second Affiliated Hospital of Zhejiang University School of Medicine and Hangzhou First People’s Hospital between March 2022 and February 2023. Implantation site localization was determined using the 18-segment method under RAO 30° imaging. Intraoperative and 1-month post-operative echocardiography was performed to assess cardiac electromechanical synchronization and tricuspid regurgitation; parameters of interest included interventricular mechanical delay (IVMD), pre-ejection period of the aorta (L-PEI), and septal-to-posterior wall motion delay (SPWMD). Pacing thresholds, sensing, and impedance exhibited no significant differences between the 8/9 zone and other sites (*P* > 0.05). In contrast, the 8/9 zone group manifested a significant reduction in L-PEI (128.24 ± 12.27 vs. 146.50 ± 18.17 ms, *P* < 0.001), IVMD (17.92 ± 8.47 vs. 28.56 ± 15.16 ms, *P* < 0.001), and SPWMD (72.84 ± 19.57 vs. 156.56 ± 81.54 ms, *P* < 0.001), compared to the non-8/9 group. Post-pacing QRS duration showed no significant difference between the two groups (139.21 ± 11.36 vs. 143.83 ± 16.35 ms *P* = 0.310). Notably, for patients with atrial fibrillation, the 8/9 zone placement significantly reduced tricuspid regurgitation. During the 1-month follow-up, neither group reported major complications such as bleeding, cardiac tamponade, pacemaker detachment, or malignant arrhythmias.

**Conclusion:**

Implantation of the leadless pacemaker in the right ventricular 8/9 zone provides superior electromechanical synchronization compared to other sites.

## Background

The advent of the leadless pacemaker, a revolutionary device that integrates a pulse generator with pacing electrodes, has effectively overcome complications associated with the pouch and leads characteristic of conventional pacemakers. This innovation not only enhances patient safety but also significantly improves their quality of life [1]. However, despite its advantages, this pacemaker is not suitable for His-bundle pacing. In patients with high pacing demands, it may disrupt the natural ventricular activation sequence, leading to cardiac dyssynchrony. This not only amplifies sympathetic nerve activity but can also trigger pacemaker-induced cardiomyopathy (PICM), which can impair the heart’s contractile function, culminating in cardiac dysfunction [2, 3].

Prior studies indicate that pacing location can influence the breadth and synchrony of the QRS complex [4]. Furthermore, right ventricular pacing that induces dyssynchrony between the ventricles is correlated with an increased prevalence of tricuspid valve insufficiency [5]. In light of this, our study leverages cardiac ultrasonography to meticulously assess the effects of various implantation sites of the leadless pacemaker on cardiac synchrony and tricuspid valve function. Our objective is to identify the optimal implantation site, offering crucial insights for future leadless pacemaker placements.

## Methods

### Study population

This dual-center clinical trial, conducted from March 2022 to February 2023, enrolled 53 patients from the Second Affiliated Hospital of Zhejiang University School of Medicine and the First People’s Hospital of Hangzhou. Inclusion criteria required patients to consent to the implantation of a leadless pacemaker. Exclusion criteria were: 1) presence of inferior vena cava or portal vein thrombosis, tumor thrombus, or inferior vena cava malformation; 2) femoral vein stenosis or tortuosity preventing the accommodation of the pacemaker delivery system; 3) patients in the acute phase of myocardial infarction; 4) patients with implanted devices that interfere with the pacemaker delivery system, such as inferior vena cava filters or mechanical tricuspid valve replacements; and 5) patients unable to comprehend or unwilling to complete an informed consent form for follow-up. The patients were assigned to two groups according to the implantation site of their leadless pacemaker for intergroup comparisons.Ethical considerations related to this study underwent thorough review and approval by the Ethics Committee of the two hospitals. All patients provided written informed consent.

### Implantation of leadless pacemaker

The leadless pacemaker implantation procedure is performed under local anesthesia. Ultrasound guidance is used to puncture the femoral vein, followed by the insertion of an 11F femoral vein sheath. A femoral venogram is performed to assess the anatomy of the vasculature and upon adequate dilation of the puncture site, a guidewire is advanced to the right subclavian vein. Subsequently, the delivery sheath is advanced over the guidewire to the middle of the atrium, following which the guidewire and inner sheath are removed.

Following this step, the delivery sheath undergoes aspiration and flushing, is connected to heparinized saline infusion, and the delivery system is inserted into the sheath, advancing into the atrium. The delivery system is maneuvered across the tricuspid annulus, and the catheter is advanced to the target position in the right ventricle. After angiographic confirmation of the position, sustained forward pressure is applied against the ventricular wall as the device is deployed from the delivery tool.

Subsequently, the delivery system is retracted from the device, and pacemaker-related parameters are tested, including a pull-test where the tether is gently pulled, and the high-magnification image is observed for at least two tines securing in place, recording for a continuous 10 s. Pacing threshold, *R*-wave amplitude, and impedance are re-evaluated.

Once the parameters are confirmed satisfactory, the tether is cut under imaging to ensure its movement does not affect the device’s fixation. The delivery sheath is removed, and images in the AP, RAO 30°, and LAO 45° positions are retained. The femoral vein puncture site is closed with a figure-eight suture and dressed with gauze to stop bleeding.

### Site determination methods under radiological guidance for 9 and 18 sections

**Fig. 1:**
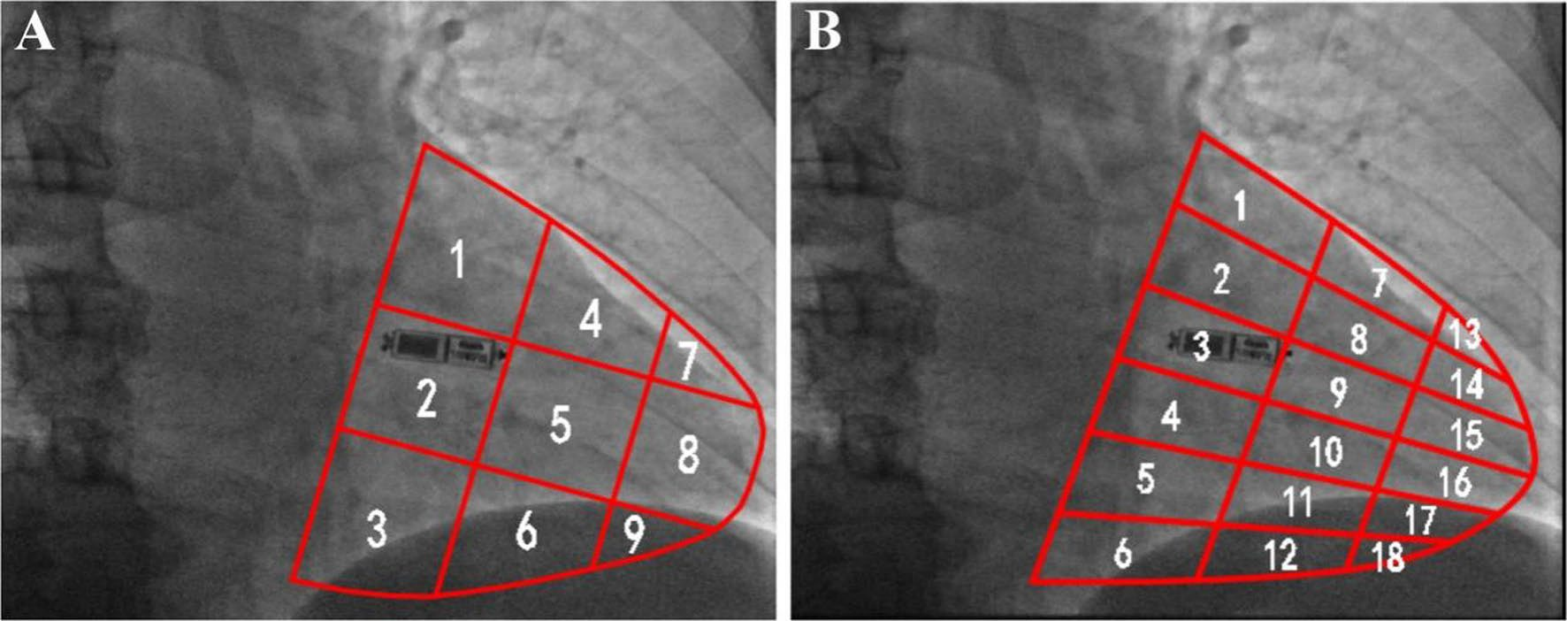
Methods of localization under radiological guidance for 9 and 18 sections. **A** Image diagram of the nine-zoning method. **B** Image diagram of the 18-zoning method

For 9 sections, the procedure involves imaging patients at RAO 30°. The ventricular contraction ring serves as the base, with the apex of the heart as the vertex. This arrangement vertically divides the heart into three equal parts, creating the upper, middle, and lower segments of the interventricular septum. Horizontally, the heart is divided into three equal parts—the anterior, middle, and posterior segments of the interventricular septum. The overlap of these two divisions divides the ventricle under the RAO 30° projection into 9 regions (Fig. 1A) [6].

For 18 sections, an extension of the nine-section approach involves horizontal division into six equal parts, yielding a total of 18 regions (Fig. 1B).

### Follow-up and relevant evaluation indicators

For all study participants, comprehensive assessments of pacing electrophysiological parameters were conducted by recording changes in pacing threshold, pacing perception, and pacing impedance both during and after the leadless pacemaker procedure. The goal was to evaluate the stability of these parameters over time.

Echocardiography was employed to measure key indicators, including QRS width pre- and post-pacing, interventricular mechanical delay (IVMD), pre-ejection period of the aorta (L-PEI), and septal-to-posterior wall motion delay (SPWMD). These metrics provided valuable insights into the mechanical synchronization of the heart. Specifically, IVMD values of 40 ms or higher, or L-PEI values of 140 ms or more, were considered indicative of desynchronization between the ventricles. An SPWMD value exceeding 130 ms signified intraventricular desynchronization [7, 8].

Additionally, color Doppler dynamic echocardiography was employed to measure the vena contracta (VC) and effective regurgitant orifice area (EROA), both before and after pacing. This helped determine the severity of tricuspid regurgitation. Based on our measurements, tricuspid regurgitation was classified into five levels: decreased by two levels, decreased by one level, no change, worsened by one level, and worsened by two levels. An increase of at least one level post-pacing was categorized as a progression in tricuspid regurgitation.

### Statistical analysis

Data analyses were performed using SPSS 25.0 software. Normally distributed data are presented as mean ± standard deviation (SD), and intergroup comparisons were conducted using the independent sample *t* test. Categorical data are expressed as percentages, and intergroup comparisons were carried out using the χ2 test. A *p* value less than 0.05 was considered statistically significant.

## Results

### Patient baseline characteristics

The study included 53 patients who successfully underwent leadless pacemaker implantation. The primary indications for pacemaker placement were atrial fibrillation with a slow ventricular rate (*n* = 15), sick sinus syndrome (*n* = 27), and atrioventricular block (*n* = 26). Patients were divided into two groups based on the implantation site of the leadless pacemaker: the 8/9 zone group (*n* = 37) and the non-8/9 zone group (*n* = 16). A detailed overview of comprehensive baseline clinical data is presented in Table 1.

**Table 1:** Patients baseline characteristics

Population characteristics	Patients(*N* = 53)
Age, year, mean ± SD	68.85 ± 17.93
BMI, kg/m^2^, mean ± SD	23.53 ± 3.88
Male, *n* (%)	32 (60.4)
Coronary heart disease, *n* (%)	6 (11.3)
hypertension, *n* (%)	28 (52.8)
diabetes, *n* (%)	14 (26.4)
Sick sinus syndrome, *n* (%)	27 (50.9)
Atrial fibrillation, *n* (%)	15 (28.3)
High grade AV block, *n* (%)	26 (49.1)

Data are expressed as mean ± SD or as number (percentage)

### Pacing electrophysiological parameters

All patients underwent a follow-up evaluation 1 month after surgery. Assessment of pacing thresholds, sensing, and impedance from the time of surgery to the 1-month follow-up revealed no statistically significant differences, indicating the stability of pacing electrophysiological parameters (Table 2).

**Table 2:** Pacemaker follow-up parameters

Variable (Mean ± SD)	During surgery (*N* = 53)	1-month post-op (*N* = 53)	*P* value
Sensing mV	10.26 ± 4.35	11.03 ± 4.79	0.388
Threshold V/0.4 ms	0.44 ± 0.16	0.47 ± 0.28	0.421
Impedance Ω	762.77 ± 249.19	740.00 ± 213.78	0.615

### Echocardiographic parameters

In terms of ventricular synchronization, analysis using the 18-segment method revealed statistically significant disparities in the L-PEI, IVMD, and SPWMD metrics between the 8/9 zone and non-8/9 zone groups. These findings suggest that, guided by the 18-segment approach, the 8/9 zone demonstrates superiority in synchronizing both interventricular and intraventricular contractions (Table 3).

**Table 3:** Echocardiographic parameters of cardiac synchronization

	18-segment method		
	8/9 zone (*N* = 37)	non-8/9 zone (*N* = 16)	*P* value
L-PEI	128.24 ± 12.27	146.50 ± 18.17	0.001*
SPWMD	72.84 ± 19.57	156.56 ± 81.54	<0.001*
IVMD	17.92 ± 8.47	28.56 ± 15.16	0.002*
QRS wave duration	139.21 ± 11.36	143.83 ± 16.35	0.310

Used Paired Student’s t test

*p < 0.05

Regarding tricuspid valve regurgitation, no significant statistical difference was observed in all patients before and after pacing (Table 4). However, a more nuanced analysis indicated the progression of tricuspid regurgitation after pacing in eight patients. Of these, two were from the 8/9 zone, while six were from the non-8/9 zone. Notably, a marked statistical difference existed between the 8/9 zone and non-8/9 zone groups concerning changes in tricuspid regurgitation before and after pacing (*P* = 0.003) (Table 5). For patients with atrial fibrillation, the 8/9 zone leadless pacemaker improved tricuspid valve regurgitation, but no statistical significance was found in this study (Table 6).

**Table 4:** Echocardiographic parameters of tricuspid regurgitation

	Before pacing (%)	After pacing (%)	*P* value
Tricuspid regurgitation			0.126
Mild TR	31 (58.49)	29 (54.72)	
Mild to moderate TR	3 (5.66)	8 (15.09)	
Moderate TR	10 (18.87)	14 (26.42)	
Moderate to severe TR	4(7.55)	1 (1.89)	
Severe TR	5 (9.43)	1 (1.89)

**Table 5:** Degree of change in tricuspid regurgitation after pacing compared to before pacing

Degree of change number	8/9 zone (*n* = 37)	Non-8/9 zone (*n* = 16)	*P* value
Decreased by 2 levels	6 (16.22)	2 (12.50)	0.729
Decreased by 1 levels	6 (16.22)	0 (0.00)	0.087
No change	23 (62.16)	8 (50.00)	0.409
Worsened by 1 level	2 (5.41)	3 (18.75)	0.127
Worsened by 2 levels	0 (0.00)	3 (18.75)	0.007*

Used Chi-square test

*p < 0.05

**Table 6:** Degree of change in tricuspid regurgitation after pacing compared to before pacing for patients with atrial fibrillation

Degree of change number	8/9 zone (*n* = 11)	Non-8/9 Zone (*n* = 4)	*P* value
Decreased by 2 levels	4(0.36)	2(0.50)	0.633
Decreased by 1 levels	2(0.18)	0 (0.00)	0.359
No change	5 (0.45)	1(0.25)	0.475
Worsened by 1 level	0(0.00)	1(0.25)	0.086
Worsened by 2 levels	0 (0.00)	0 (0.00)	–

### Perioperative complications

During the perioperative period, none of the enrolled patients encountered serious complications such as cardiac perforation, malignant arrhythmias, or embolism during surgery.

## Discussion

This study marks a significant step forward in utilizing the 18-segment method to assess the impact of different pacing sites on cardiac synchronization and tricuspid regurgitation. Our findings demonstrate that pacing in the 8/9 zone using a leadless pacemaker significantly enhances cardiac synchronization compared to other zones. Moreover, the complication rate associated with leadless pacemakers was remarkably low, and subsequent observations indicated the stability of pacemaker-related electric parameters. Notably, while there was no discernible statistical difference in tricuspid regurgitation before and after pacing, a noticeable reduction in regurgitation post-pacing was observed in patients with atrial fibrillation who received leadless pacemakers placed in the 8/9 zone.

Traditional cardiac pacemakers can interfere with the heart’s natural activation sequence, potentially leading to synchronization issues. Some studies suggest that pacing from the upper septal area of the right ventricle may be superior to apical pacing, although this claim has been a topic of debate [4, 9, 10]. Left bundle branch (LB) pacing or septal pacing are emerging forms of ventricular pacing, which have been proved could produce physiological electrical and mechanical activation of the left ventricle. However, there are limitations and complications should not be overlooked, such as technical complexity, late occlusion thresholds, septal perforation, and left ventricular thrombosis [11–13]. Hence, further studies are vital to confirm the long-term effectiveness of both techniques. Herein, we adopted the nine-segment methodology and identified the juncture between zones 4 and 5 as optimal for synchronization, albeit lacking precision [6]. Introducing the 18-segment method, we pinpointed the 8/9 zone as the best pacing region for heart synchronization. This zone’s proximity to the His-bundle in the high septal region offers advantages for capturing the heart’s conduction tissue, leading to better synchronization of electrical conduction between the left and right ventricles and resulting in coordinated ventricular contraction and relaxation.

While previous studies have used SPWMD, IVMD, and L-PEI as stand-alone predictors for assessing ventricular synchronization [14–16], our study amalgamated these markers to gain a comprehensive perspective on the influence of leadless pacemaker placement in diverse sites on cardiac synchronization. Although both the nine-segment and 18-segment techniques showed significant variances in SPWMD, IVMD, and L-PEI, the distinctions were more pronounced in the 18-segment approach. It is noteworthy that QRS wave parameters do not fully align with echocardiographic synchronization results, possibly due to controversies surrounding mechanical synchronization and the QRS wave [17].

The necessity for ventricular electrodes to traverse the tricuspid valve in conventional venous cardiac pacemakers increases the likelihood of tricuspid regurgitation. [18–20]. Vaidya et al. found that tricuspid regurgitation 2 months after implantation of leadless pacemakers was diminished compared to their traditional counterparts [21]. Our findings concur, demonstrating no increased tricuspid regurgitation as a result of installing leadless pacemakers in various sites. Importantly, for patients with atrial fibrillation, leadless pacemakers in the 8/9 zone improved tricuspid regurgitation. This may be related to the variable ventricular rate and annular reduction in patients with atrial fibrillation during pacing.

## Limitations

The study was constrained by a modest sample size and a brief observation period. To gain a more comprehensive understanding, future studies with a larger sample size and extended observation period are essential.

## Conclusions

The precise placement of a leadless pacemaker in the 8/9 zone, guided by the 18-segment method, might enhanced cardiac synchronization and mitigated the risk of tricuspid regurgitation. This finding likely offers valuable insights for clinical practices, particularly in the management of atrial fibrillation patients.

## Data Availability

Data are available from the authors on request.

## Abbreviations

IVMD: Interventricular mechanical delay
L-PEI: Pre-ejection period of the Aorta
SPWMD: Septal-to-posterior wall motion delay
